# Special Issue: Friction, Corrosion and Protection of Material Surfaces

**DOI:** 10.3390/ma16186220

**Published:** 2023-09-15

**Authors:** Cunao Feng, Zhenyu Wang, Zhaolei Li, Xiaowei Li

**Affiliations:** 1 School of Materials Science and Physics, China University of Mining and Technology, Xuzhou 221116, China; cunaofeng@cumt.edu.cn; 2 Key Laboratory of Marine Materials and Related Technologies, Zhejiang Key Laboratory of Marine Materials and Protective Technologies, Ningbo Institute of Materials Technology and Engineering, Chinese Academy of Sciences, Ningbo 315201, China; wangzy@nimte.ac.cn; 3 Department of Polymer Materials, School of Material Science and Engineering, Jiangsu University of Science and Technology, Zhenjiang 212100, China; zlli@just.edu.cn

The current Special Issue, entitled “Friction, Corrosion and Protection of Material Surfaces”, aims to discuss the state-of-the-art research progress regarding the friction and corrosion behaviors of new materials and advanced protective materials or technologies, with a special focus on the understanding of underlying friction and corrosion mechanisms and modification approaches of material surfaces against friction and corrosion in order to guide the design and preparation of materials with high performance for practical applications.

Friction and corrosion, which exist widely in engineering instruments, marine equipment, aerospace settings, medical equipment and other advanced manufacturing fields, are the key factors that cause surface damage to material surfaces (metals, inorganic non-metals, polymers and composite materials, such as titanium alloys, stainless steel, alumina ceramics, polyethylene, fiber-reinforced composite materials, etc.) and the failure of equipment. According to incomplete statistics, they cause billions in economic losses every year. Therefore, the friction, corrosion and protection of materials are hotspots in scientific research [1,2].

Friction and corrosion are phenomena of surface–interface interactions that are closely related to the environmental conditions in which materials and components are used. The design of materials and components should take into consideration the specific operating environment, so as to reduce the energy dissipation and component damage caused by surface corrosion and interfacial friction between interacting materials [3]. The reasonable use of lubricants or inhibitors is the most commonly used method to reduce friction or corrosion. In particular, due to their the low melting point, low vapor pressure, adjustable polarity, safety and stability, ionic liquids are used as a potentially efficient and versatile lubricants [4]. In addition, the use of two new technologies, superslippery and superlubricity [5,6], which can effectively reduce friction, is currently a key research topic. Superslippery and superlubricity allow objects to move on a surface under an ultralow friction force, and they are applicable to different application scenarios. Superslippery mainly refers to liquid–solid interfaces, while superlubricity mainly refers to solid–solid interfaces. Another well-known method is to modify and protect the surface of the material without changing its volume characteristics, which is also an effective way to alleviate corrosion, friction and wear in engineering applications. In recent years, the surface protection of traditional materials using techniques such as laser cladding, nitriding treatment, high-performance thin films and biomimetic superhydrophobic surface structure coatings has gradually replaced the use of expensive, high-performance materials and aroused much research interest [7]. Furthermore, with the development of nanotechnology, surface protective coatings for functional materials, such as nanoparticles, graphene, and diamond-like carbon materials, can be easily developed to explore their functions in reducing surface chemical/physical damage, thereby improving the performance and service life of industrial mechanical components [8,9].

The research interest of this Special Issue thus includes, but is not limited to, friction and corrosion behaviors of new materials, advanced protective materials, and advanced protective technologies. Moreover, the experimental and theoretical studies of the friction and corrosion mechanisms and advances in the friction, corrosion and protection of material surfaces are the central topics of this Special Issue.
